# Mechanical Properties of Aramid/Carbon Hybrid Fiber-Reinforced Concrete

**DOI:** 10.3390/ma14195881

**Published:** 2021-10-08

**Authors:** Yeou-Fong Li, Hsin-Fu Wang, Jin-Yuan Syu, Gobinathan Kadagathur Ramanathan, Ying-Kuan Tsai, Man Hoi Lok

**Affiliations:** 1 Department of Civil Engineering, National Taipei University of Technology, Taipei 10608, Taiwan; student6101@gmail.com (H.-F.W.); t9679010@ntut.org.tw (J.-Y.S.); gobiram0017@gmail.com (G.K.R.); 2 Department of Environmental Information and Engineering, Chung Cheng Institute of Technology, National Defense University, P.O. Box 90047-82, Dasi, Taoyuan 33550, Taiwan; ccitb04007@ndu.edu.tw; 3 Department of Civil and Environmental Engineering, Faculty of Science and Technology, University of Macau, Taipa, Macau, China; mhlok@um.edu.mo

**Keywords:** aramid fiber, carbon fiber, concrete, compressive strength, flexural strength, splitting strength, impact energy

## Abstract

In this study, aramid fiber (Kevlar^®^ 29 fiber) and carbon fiber were added into concrete in a hybrid manner to enhance the static and impact mechanical properties. The coupling agent presence on the surface of carbon fibers was spotted in Scanning Electron Microscope (SEM) and energy-dispersive X-ray spectroscopy (EDS) graphs. The carbon fiber with a coupling agent affected the mechanical strength of the reinforced concrete. At 1% fiber/cement weight percentage, the hybrid fiber-reinforced concrete (HFRC) prepared using Kevlar fiber and carbon fiber of 12 and 24 mm in length under different mix proportions was investigated to determine the maximum mechanical strengths. From the test results, the mechanical strength of the HFRC attained better performance than that of the concrete with only Kevlar or carbon fibers. Foremost, the mix proportion of Kevlar/carbon fiber (50–50%) significantly improved the compressive, flexural, and splitting tensile strengths. Under different impact energies, the impact resistance of the HFRC specimen was much higher than that of the benchmark specimen, and the damage of the HFRC specimens was examined with an optical microscope to identify slippage or rupture failure of the fiber in concrete.

## 1. Introduction

Concrete is the most widely used material in construction for civil and military purposes, but it has low tensile strength, poor fracture toughness, and is susceptible to brittle failure. Under repeated loadings, concrete is easily prone to cracks or damage, which might reduce the service life of the concrete structures. To enhance the mechanical performance of the concrete, fibers are usually added into concrete to improve the toughness, tensile properties, impact resistance, and fatigue durability.

In fiber-reinforced concrete (FRC) structures, polypropylene, steel, glass, basalt, carbon, and aramid fibers are commonly added to improve the toughness. Compared with polypropylene fiber, steel fiber has higher tensile strength and elastic modulus. Adding steel fibers to concrete can improve the compressive strength and toughness of concrete [1,2,3,4]. With the addition of a 1.5% volume fraction of steel fiber in concrete, steel fiber-reinforced concrete has the greatest compressive and flexural strengths [5,6].

Glass fiber is a high-strength artificial fiber. Adding glass fiber to concrete can improve the flexural and splitting strength of concrete. With the increase in glass fiber content, the effect of improving concrete toughness and restraining concrete fracture is evident [7,8,9,10]. However, glass fiber is not alkali resistant, and long-term alkali corrosion will reduce its strength. Adding a 2.5% fiber weight fraction of alkali-resistant glass fiber to lightweight concrete can increase the maximum flexural strength and inhibit the expansion of concrete cracks [11]. Basalt fiber has good resistance to acid and alkali solutions, as well as high strength and high-temperature resistance. The effect of adding basalt fiber into the concrete mixture to improve the compressive strength is not evident, but it can increase the flexural and splitting strength [12,13,14,15].

Carbon fiber is a high-strength and lightweight fiber. It has the characteristics of fatigue resistance, ultra-high temperature resistance, and corrosion resistance. Adding carbon fiber to concrete can improve mechanical strength and flexural performance and inhibit crack propagation [16,17,18,19,20,21,22]. Aramid fiber is an organically synthesized high-tech fiber, which has the characteristics of high tensile strength, low weight, high abrasion resistance, high impact resistance, and high energy absorption. Incorporating aramid fiber into concrete can improve the compressive strength of concrete [23,24].

Hybrid fiber-reinforced concrete (HFRC) is a new trend in concrete research. When two or more kinds of fibers are added to concrete simultaneously, combining different types of fiber enhances the mechanical strength compared with single fiber reinforced concrete [25,26,27,28,29,30]. When steel fibers and polypropylene fibers are added to concrete, steel fiber provides higher strength, while polypropylene fiber can inhibit the growth of cracks in the concrete. Both the mechanical strength and impact resistance of HFRC are much higher than that of concrete containing only steel fibers or polypropylene fibers [31,32,33]. Additionally, when basalt fiber and polypropylene fiber are added into concrete, the compressive, flexural, and splitting strength of the HFRC are also higher than that of concrete containing only one kind of fiber [34,35]. Compared with different HFRCs, the mechanical strength of hybrid steel fiber and basalt fiber HFRC is higher than that of hybrid steel fiber and polypropylene fiber, or hybrid basalt fiber and polypropylene fiber HFRC [36]. It has been found that adding fibers to concrete can improve the mechanical strength, toughness, and crack resistance of concrete. At the same time, adding hybrid fibers can greatly enhance the static and dynamic mechanical properties of concrete, and the effect is more pronounced than adding only one fiber. Hybrid fiber-reinforced concrete can be applied to rigid pavements, expansion joints, airport runways, bridge piers, tunnels, dams, slope reinforcements, and other concrete structures that bear repeated loads and impacts for a long period of time. Hybrid fiber-reinforced concrete can also achieve enhanced ability to resist shock waves and can be used in hangars, refuges, chemical plants, etc. The high strength and high elongation of fiber can improve the static and dynamic mechanical properties of fiber-reinforced concrete. Aramid fiber and carbon fiber have high strength, low density, and good weather resistance, unlike glass fiber and steel fiber that are vulnerable to alkaline environments or rust [37,38]. At the same time, aramid fiber has good elongation, which can inhibit the expansion of concrete cracks.

Li et al. [39] studied concrete reinforced with different lengths of carbon fiber in flexural, compressive, and impact tests. The experimental results of 12 and 24 mm carbon fiber reinforced concrete attained the highest flexural strength and impact resistance compared with those of 6 mm. The strength of concrete reinforced with carbon fiber without a coupling agent is better than that reinforced with carbon fiber with a coupling agent, and that with the fiber/cement ratio of 1% has the highest strength [40,41]. The strength of Kevlar fiber with a coupling agent is higher than that of Kevlar fiber without a coupling agent. The 12 mm Kevlar fiber and 24 mm Kevlar fiber-reinforced concrete show similar strength, and also the fiber/cement ratio of 1% has the highest strength [42]. Therefore, the hybrid fiber mix proportion (Kevlar/carbon) of 1% weight percentage was used in this research study. The Kevlar fiber with a coupling agent and the carbon fiber without a coupling agent were used, and then fibers were incorporated into the concrete to determine the strength in the mix proportions of Kevlar (12 mm)/carbon (24 mm) and carbon (12 mm)/Kevlar (24 mm).

In this study, aramid and carbon fibers were chopped into 12 and 24 mm lengths. Seven different mix proportions were mixed into concrete under 1% fiber/cement weight percentage to determine the compressive, flexural, and splitting strengths, as well as impact resistance. In addition, the pneumatic method was used to disperse the fibers. Aramid fiber/carbon fiber hybrid fiber-reinforced concrete (HFRC) was tested at different mix proportions to determine the maximum strength.

## 2. Materials and Experimental Methods

### 2.1. Materials

Carbon and aramid fibers have a higher tensile strength of elasticity than other fibers, so they can assist the fiber-reinforced concrete (FRC) to withstand the load by enhancing the tensile strength. Carbon and aramid fibers were chopped into 12 and 24 mm lengths, respectively, and added into concrete at different mix proportions. For each fiber to be effective, it is necessary to use aerodynamic force to disperse the fibers.

#### 2.1.1. Cement

The Portland cement Type I was used, which was obtained from Taiwan Cement Corporation (Taipei, Taiwan).

#### 2.1.2. Aggregate

The standard specification and fineness modulus (FM) of concrete aggregate is based on ASTM C33/C33M-18 [43], the FM of fine aggregates is 3.03, and of coarse aggregates is 7.33. The FM of all aggregates is 5.96.

#### 2.1.3. Kevlar Fiber

Kevlar^®^ 29 is one of the aramid fibers, and it is a lightweight material with high tensile strength, high modulus elasticity, and high fracture toughness. The Kevlar^®^ 29 fiber is obtained from the Dupont Company. Kevlar fiber is often used in military equipment such as ballistic helmets and ballistic vests, marine ropes, fire-resistant equipment, and tire reinforcement materials [44]. It has heat-resistant and chemically stable qualities that can withstand a wide range of chemicals, including acetic acid, hydrochloric acid, and other solvents. The material properties of Kevlar^®^ 29 and carbon fibers are shown in Table 1.

#### 2.1.4. Carbon Fiber

The Polyacrylonitrile (PAN) based carbon fibers were obtained from Tairylan Division, Formosa Plastics Group, Kaohsiung, Taiwan. The PAN based carbon fiber was made by the spinning process, thermal stabilization, and carbonization stages. Carbon fiber has a low specific density, high strength, high fatigue resistance, and high temperature and corrosion resistance. It is often used in the aviation industry, sports equipment, wind turbines, transportation, and other applications. The material properties of carbon fiber are also shown in Table 1.

**Figure 1:**
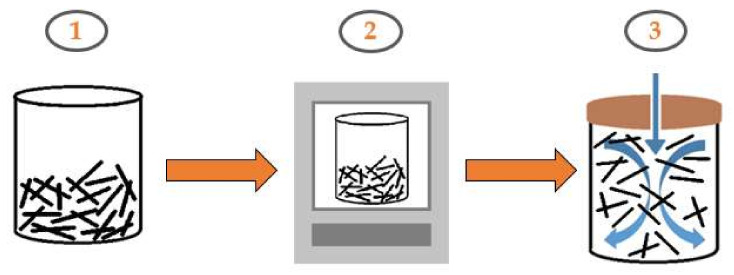
Chopped carbon fiber coupling agent removal process: (**1**) chopped carbon fiber; (**2**) carbon fiber heated at 550 °C (muffle furnace); (**3**) pneumatic dispersion.

To improve the strength of concrete, it is necessary to remove the coupling agent from the surface of the carbon fibers. The process of removing the coupling agent requires the fibers to be heated in a muffle furnace at a high temperature (550 °C) for 3 h, as shown in Figure 1.

#### 2.1.5. Hybrid Fiber-Reinforced Concrete (HFRC)

**Figure 2:**
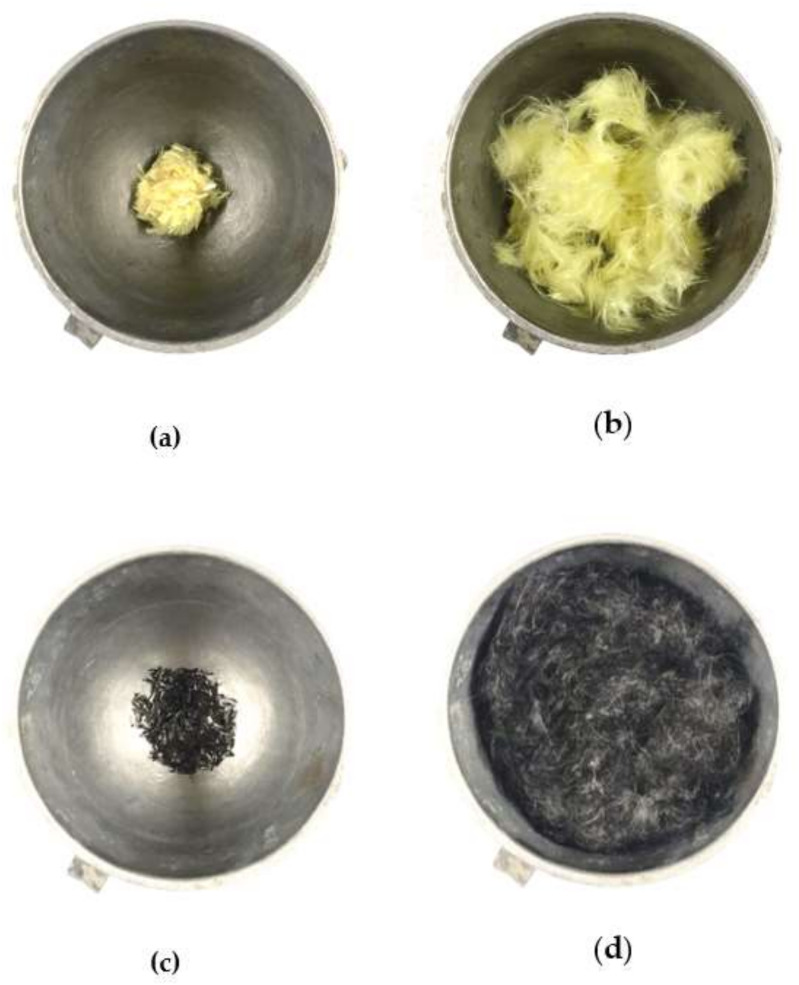
The pneumatic dispersion process of Kevlar fiber and carbon fiber: (**a**) Kevlar fiber before pneumatic dispersion; (**b**) Kevlar fiber after the pneumatic dispersion; (**c**) carbon fiber before pneumatic dispersion; (**d**) carbon fiber after the pneumatic dispersion.

The chopped Kevlar and carbon fiber were dispersed by the pneumatic (aerial) method. The pneumatic dispersion process of Kevlar fiber and carbon fiber is shown in Figure 2. In the dry state, the dispersed fibers are mixed with the cement in an evenly distributed manner, and they are finally mixed with the aggregates and water in wet conditions to prepare the HFRC.

The mix-ratio of the cement, sand, and aggregates were 1:1.05:2.25 and the water-cement ratio is 0.6. In the following tests, the chopped Kevlar and carbon fibers were added to the concrete specimens by 1% weight percentage of cement. The first percentage stands for 12 mm fiber, and the second percentage stands for 24 mm fiber. Seven different mix proportions were used in this study:100–0%, 80–20%, 60–40%, 50–50%, 40–60%, 20–80%, and 0–100%.

### 2.2. Experimental Methods

The compressive, flexural, and splitting tensile tests were conducted following ASTM standards, and the impact test was conducted following ACI standards. Table 2 shows the preparation planning of compressive, flexural, and impact tests for the benchmark and hybrid fiber-reinforced concrete (Kevlar/carbon) specimens under different mix proportions.

#### 2.2.1. SEM and EDX

Tabletop microscopes (TM4000plus II, Hitachi High-Tech Corporation, Tokyo, Japan) were used to examine the surface of the carbon fiber and analyze the element content. The SEM image shows the surface observation of the carbon fiber at 1000 times magnification, and the EDX graph shows the percentage of coupling agent on the chopped carbon fiber.

#### 2.2.2. Slump Test

The fiber weight percentage and water/cement ratio affect the workability of reinforced concrete. Adding fibers to the concrete in high volume content and weight percentages leads to reduced workability of concrete. In accordance with ASTM C143/C143M-20 [45], the slump consistency (15 mm~230 mm) was analyzed by FRC at a 1% weight percentage.

#### 2.2.3. Compressive Test

The compressive strengths of HFRC specimens were determined according to ASTM C39/C39M-01 [46], and the benchmark and HFRC specimens were examined with different mix proportions. The compressive test of cylindrical specimens was conducted in the universal test machine with the dimensions of φ10 cm × 20 cm, and the loading rate of 66~160 kN/min.

The compressive, flexural, and splitting tensile strengths were determined using the universal testing machine (HT-9501 Series. Hong-Ta, Taipei, Taiwan), with a load cell (WF 17120, Wykeham Farrance, Milan, Italy) at the laboratory of the Department of Civil Engineering, National Taipei University of Technology.

#### 2.2.4. Three-Point Bending Test

In accordance with ASTM C293/C293M-16 [47], the benchmark and HFRC specimens were subjected to the three-point bending test, and the flexural strength was obtained at a loading rate of 0.020 MPa/s. The dimensions of the HFRC specimen are 28 cm × 7 cm × 7 cm (length × width × height).

*R* = 3*PL*/2*bd*^2^ (1)

In Equation (1), *R* is the flexural strength (MPa); *P* is the maximum applied load (N); *L* is span length (mm); *b* is the average width of the specimen (mm); and *d* is the average depth of specimen (mm).

#### 2.2.5. Splitting Tensile Test

The splitting tensile test of HFRC specimens was conducted according to ASTM C496/C496M-17 [48]. The splitting tensile strengths of cylindrical specimens were determined using the universal test machine with the loading rate of 22~44 kN/min and the dimensions of φ10 cm × 20 cm.

In Equation (2), *T* is the splitting tensile strength (MPa); *P* is the maximum applied load indicated by the testing machine (N); *L* is the length of the specimen (mm); and *D* is the diameter of the specimen (mm).

*T* = 2*P*/*πLD* (2)

#### 2.2.6. Impact Test

**Figure 3:**
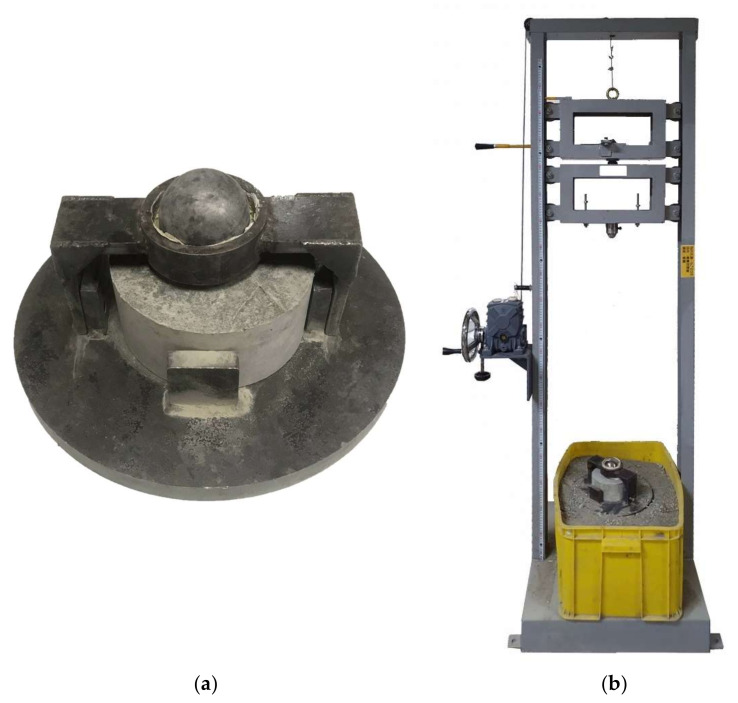
Free-fall impact test: (**a**) test equipment and KFRC specimen; (**b**) test machine.

In accordance with ACI 544.2R-89 [49], the HFRC specimens were tested, and the impact numbers under different impact energies were determined. The dimensions of the benchmark and HFRC specimens are ϕ 15.2 cm × 6.35 cm, and the impact energies are 50~150 J. The impact energies were changed by increasing the weight of the projectile, and the impact test was improved. The weight of the projectile was 15 kg~27.4 kg, and then the projectile was hit on the steel ball on the surface of the concrete specimens. The benchmark and HFRC specimens were tested at different impact energies, as the height of the projectile was 0~100 cm, and the sandbox was placed under the HFRC specimen to adsorb the energy after impact. Figure 3a shows the equipment for impact strength and the concrete specimen, and Figure 3b shows the impact test equipment (SP-005, Sheng Peng Applied Materials Co., Ltd., Yu-Lin, Taiwan). The impact energy is *E = m × g × h, E* is potential energy (J), *m* is mass (kg), *g* is the acceleration of gravity (m/s^2^), and *h* is the height (m).

#### 2.2.7. Optical Microscope Surface Analysis

The failure surface of the HFRC specimen was analyzed by an optical microscope (UPG650, UPMOST, Taiwan) to examine whether the two fibers were evenly distributed and effectively adhered to the concrete, using a high magnification of 100 times.

## 3. Results and Discussion

The disparate length of Kevlar and carbon fibers are incorporated with cement to prepare HFRC specimens. The HFRC specimens compressive, flexural, and splitting tensile strength, as well as impact performance, are listed below. The specimens were prepared with a 0.6 water-cement ratio and cured for 28 days.

### 3.1. SEM and EDX Graph Results

**Figure 4:**
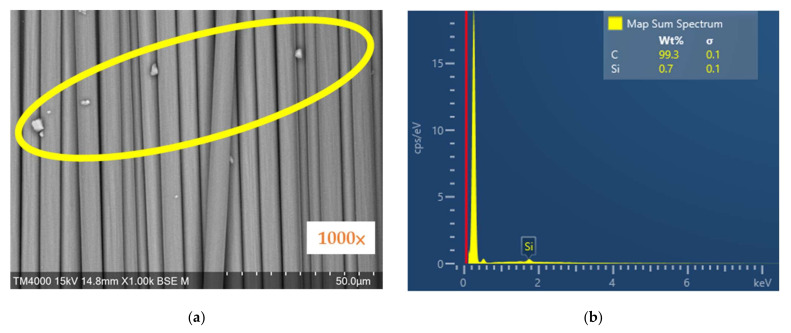
SEM observation of the surface of carbon fiber with the presence of coupling agent: (**a**) SEM image; (**b**) EDX graph.

Figure 4a shows the SEM image of carbon fiber in the presence of the coupling agent, as the sporadic grains inside the yellow oval, and Figure 4b shows the EDX analysis of carbon fiber in the presence of the coupling agent. It can be observed that the carbon content on the surface of the carbon fiber is 99.3%, and the silicon content is 0.7%.

**Figure 5:**
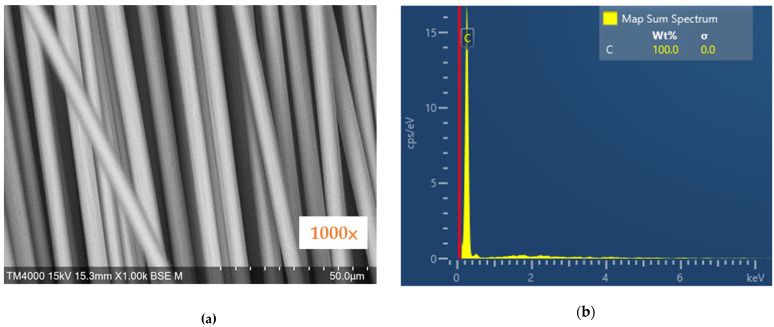
SEM observation of carbon fiber by removal of coupling agent: (**a**) SEM image; (**b**) EDX graph.

The carbon fiber without the coupling agent was examined using an SEM, and the image is shown in Figure 5a, and the EDX analysis is shown in Figure 5b. It can be observed that the carbon content on the surface of the carbon fiber is 100%. There are no other substances on the surface of carbon fiber as compared to Figure 4, and this demonstrates that high-temperature heating can effectively remove the coupling agent.

### 3.2. Slump Test Results

The slump values of benchmark and FRC mixtures with fiber/cement ratio of 1% weight percentage are shown in Table 3. The test results showed that the slump value was not affected by the carbon and Kevlar fibers, but the slump values varied under different mix proportions. The HFRC mixture slump values are between 70~80 mm in different mix proportions.

### 3.3. Compressive Test Results

The 1% fiber cement weight percentage for HFRC was used in the following tests. Seven different mix proportions—100–0%, 80–20%, 60–40%, 50–50%, 40–60%, 20–80%, and 0–100%—were prepared, and the maximum compressive strength for mix proportions of 1% Kevlar/carbon and carbon/Kevlar specimens was determined. The first percentage stands for 12 mm fiber, and the second percentage stands for 24 mm fiber.

**Figure 6:**
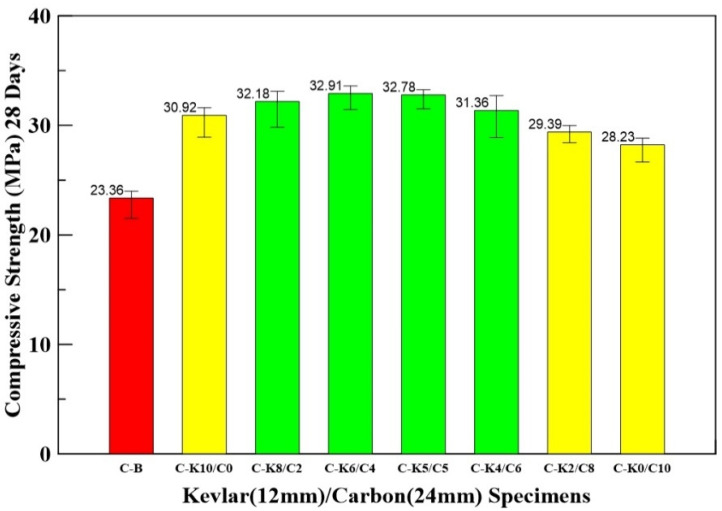
Bar chart for average compressive strengths of benchmark and Kevlar/carbon HFRC specimens.

The naming of the Kevlar/carbon HFRC specimen with different mix proportions is described as follows. Specimen C-K8/C2 is an example: the first C stands for compression, and K8/C2 stands for Kevlar fiber 80% and carbon fiber 20%. Table 4 shows the compressive strengths of benchmark and different mix-proportion Kevlar (12 mm)/carbon (24 mm) HFRC specimens. Comparing with the benchmark specimen, the compressive strengths of HFRC specimens C-K8/C2, C-K6/C-4, C-K5/C5, and C-K4/C6 increase by 38, 41, 40, and 34%, respectively. The average compressive strengths of different mix-proportion Kevlar/carbon HFRC specimens are shown in Figure 6.

**Figure 7:**
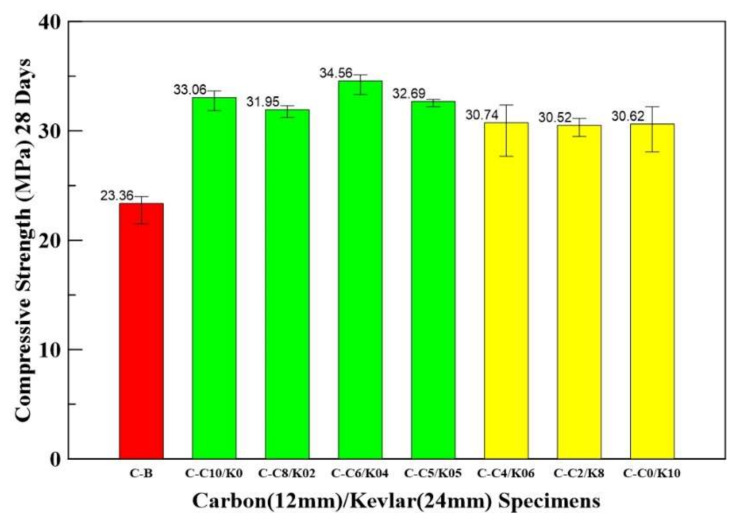
Bar chart for compressive strengths of benchmark and carbon/Kevlar HFRC specimens.

Table 5 shows the compressive strengths of benchmark and carbon (12 mm)/ Kevlar (24 mm) HFRC specimens with different mix proportions. Comparing with the benchmark specimen, the compressive strengths of HFRC specimens C-C10/K0, C-C8/K2, C-C6/K4, and C-C5/K5 increase by 41, 37, 48, and 40%, respectively. The specimen C-C6/K4 has the highest compressive strength, which increases by 48%. The average compressive strengths of benchmark and carbon/Kevlar HFRC specimens are shown in Figure 7.

### 3.4. Three-Point Bending Test Results

The 1% weight percentage for HFRC was used in the following tests. The 1% weight percentage of HFRC was exploited with different mix proportions to determine the ultimate flexural strength. The naming of the Kevlar/carbon HFRC specimen with different mix proportions is described as follows. Specimen F-K6/C-4 is an example: the F stands for flexural, and K6/C-4 stands for Kevlar fiber 60% and carbon fiber 40%.

**Figure 8:**
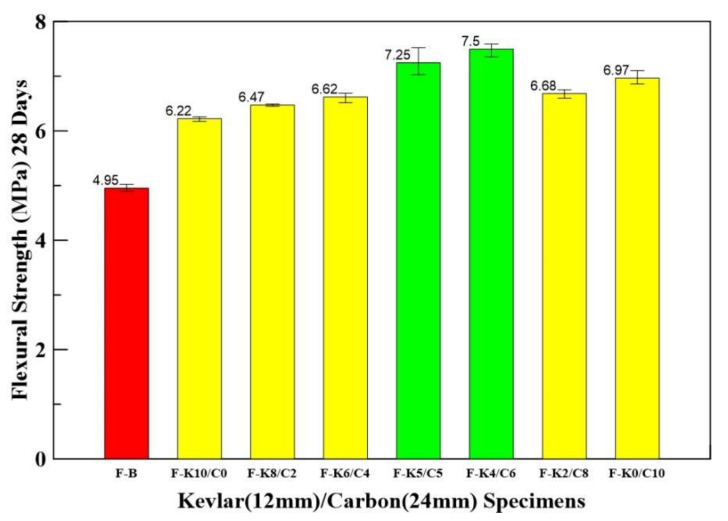
Bar chart for average flexural strengths of benchmark and Kevlar/carbon HFRC specimens.

Table 6 shows the flexural strength of Kevlar (12 mm)/carbon (24 mm) HFRC specimens with different mix proportions. The mix proportions of Kevlar/carbon HFRC specimens increase their flexural strength by 26~51% compared with benchmark specimens. The F-K5/C5 and F-K4/C6 have higher flexural strength, increasing by 46 and 51%, respectively. The flexural strengths of HFRC are shown in Figure 8.

**Figure 9:**
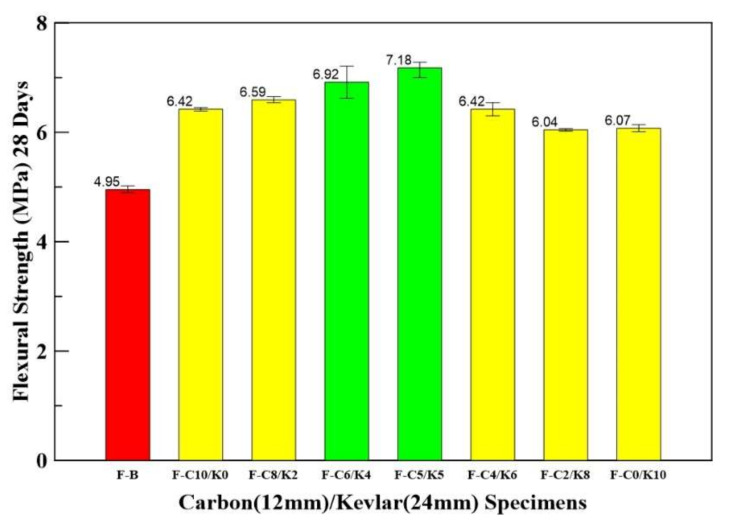
Bar chart for average flexural strengths of benchmark and carbon/Kevlar HFRC specimens.

Table 7 shows the flexural strength of benchmark and carbon (12 mm)/ Kevlar (24 mm) HFRC with different mix-proportion specimens. The mixed proportions of carbon/ Kevlar HFRC specimens increased their flexural strength by 22~45% compared with benchmark specimens. The F-C6/K4 and F-C5/K5 have higher flexural strength than others, and they increase by 40 and 45%, respectively. The average flexural strengths of HFRC are shown in Figure 9.

### 3.5. Splitting Tensile Test Results

From the above compressive and flexural test results, the HFRC specimens have better compressive and flexural strength in the 50–50% mix proportion. Therefore, the HFRC specimens in 50–50% mix proportion are used to determine the splitting tensile strength. The naming of the specimen was described as follows. Specimen S-K5/C5 is an example: the S stands for splitting tensile test, and K5/C5 stands for Kevlar fiber 50%/carbon fiber 50%.

**Figure 10:**
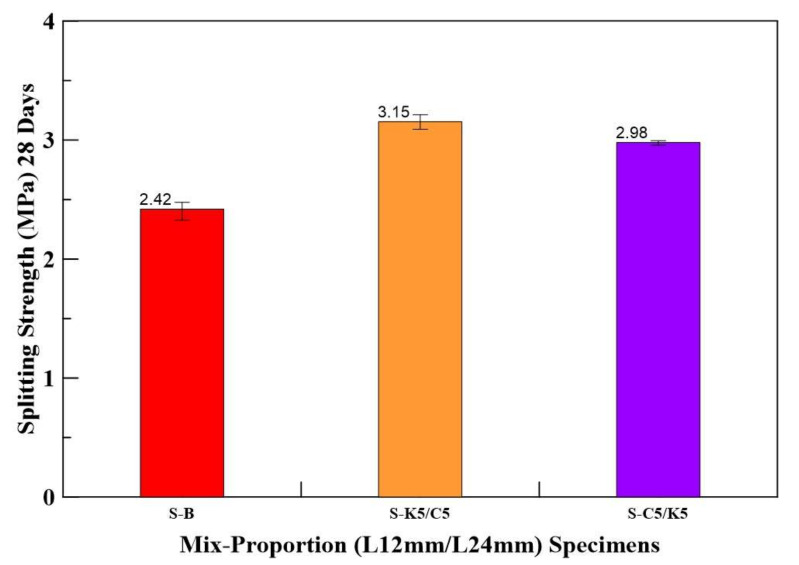
Bar chart for average splitting tensile strengths of benchmark and HFRC specimens.

The splitting tensile strength results of HFRC specimens are shown in Table 8. The splitting tensile test results show that specimen S-K5/C5 has the highest splitting tensile strength, and it is increased by 30% compared with the benchmark. The Bar chart for average splitting tensile strengths of the benchmark and the HFRC specimens are shown in Figure 10.

### 3.6. Impact Test Results

From the above test results, the HFRC specimens attained higher compressive, flexural, and splitting tensile strengths in 50–50% mix proportions. Therefore, the HFRC specimens in 50–50% mix proportions are used to determine the impact resistance.

Table 9 shows the impact numbers of benchmark and Kevlar/carbon HFRC specimens. Under 50 J impact energy, the average number of impacts at the failure of benchmark specimens was 13.5, and those for the I-K5/C5 and I-C5/K5 specimens were 538.3 and 473.8, respectively. Therefore, the I-K5/C5 specimens were stronger compared to others.

Li et al. [39] studied the impact test with 24 mm carbon fiber-reinforced concrete. Under 50 J impact energy, the average number of impacts at the failure of CFRC specimens was 409.8. The average number of impacts of 24 mm Kevlar fiber-reinforced concrete (KFRC) was 402.5 [42]. Therefore, the impact resistance of the Kevlar/carbon HFRC specimens was higher than CFRC and KFRC specimens.

**Figure 11:**
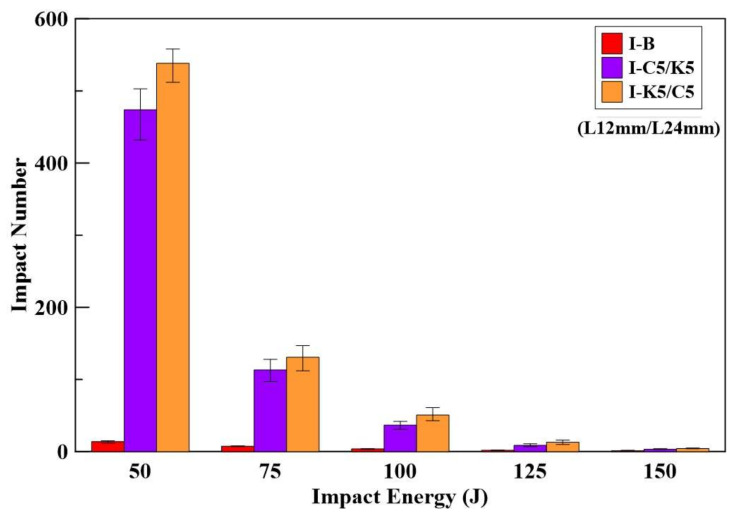
Bar chart for impact energy–impact number relationship of the benchmark and HFRC specimens.

The impact test results show that specimen I-K5/C5 has the best impact-resistant performance under different impact energies. The relationships for the number of impacts and the impact energy for both the benchmark and HFRC specimens are shown in Figure 11.

**Figure 12:**
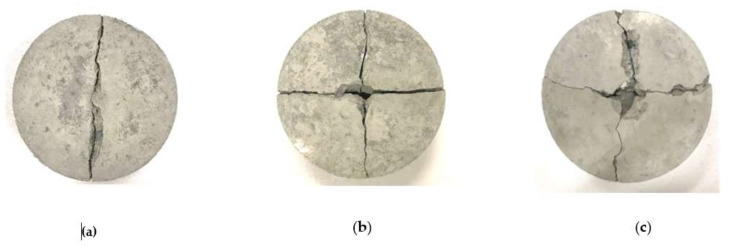
The failure photos of the specimen under repeated impact at 50 J: (**a**) I-B; (**b**) I-K5/C5; and (**c**) I-K5/C5.

The impact numbers of I-K5/C5 and I-C5/K5 specimens were higher than those of the benchmark specimen. After the impact test, the I-K5/C5 and I-C5/K5 specimens were broken into four pieces and the benchmark specimens into two pieces under repeated impacts at 50 J, as shown in Figure 12. It can be concluded that more broken pieces indicate higher impact resistance.

### 3.7. Optical Microscope

**Figure 13:**
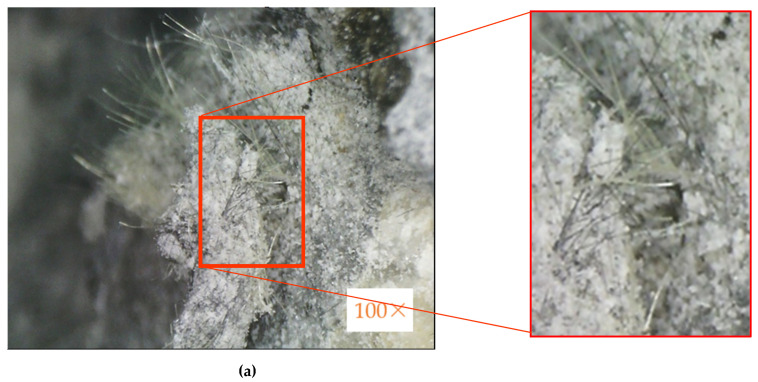

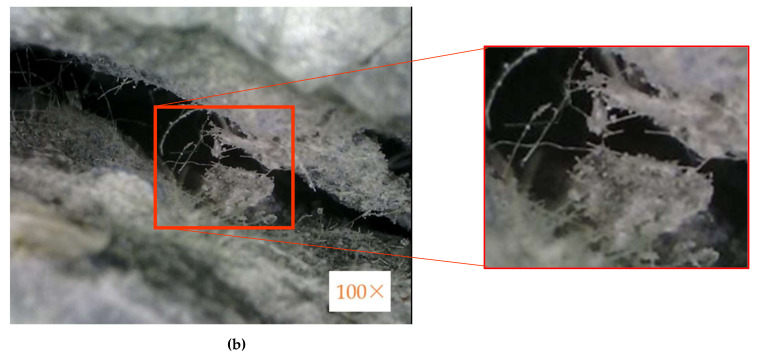
Optical microscopic image of I-K5/C5 specimen’s failure mode under impact loading: (**a**) fracture, (**b**) crack.

The impact test results indicate that the I-K5/C5 specimen had the best impact resistance, so the fracture surface of the I-K5/C5 specimen after the impact test was subjected to optical microscope surface analysis. Figure 13 shows the optical microscope image of I-K5/C5 specimens with a magnification of 100 times. Figure 13a shows (inside the red box) the Kevlar and carbon fiber were uniformly distributed, and Figure 13b shows (inside the red box) the two fibers had effectively adhered to the concrete, which can inhibit the expansion of cracks and improve the impact strength of I-K5/C5 specimens.

## 4. Conclusions

Carbon and Kevlar fibers can enhance the static and dynamic behavior of HFRC specimens under different mix proportions, and the following conclusions are listed below.

1. The results show that HFRC with different mix proportions has similar slump values by 1% weight cement percentage and between 70–80 mm.
2. The compressive test results show that the HFRC specimens attained higher compressive strength in C-C6/K4 (60–40%) and C-C5/K5 (50–50%) mix proportions. Compared with the benchmark specimen, the compressive strengths of the C-C6/K4 and C-C5/K5 specimens increased by 48 and 40%, respectively.
3. The F-K5/C5 and F-K4/C6 specimens have better flexural strength than the benchmark specimens, increasing by 46 and 51%, respectively.
4. Splitting tensile test results show that the S-K5/C5 specimen has the highest splitting tensile strength. Compared with the benchmark specimen, the S-K5/C5 specimen splitting tensile strength increased by 30%.
5. The I-K5/C5 specimen has the best impact resistance in the impact test and it resists 512~558 impact numbers under 50 J.

## Figures and Tables

**Table 1:** Material properties of Kevlar^®^ 29 and carbon fibers.

Material Property	Fiber	
	Kevlar^®^ 29	Carbon
Density (g/cm^3^)	1.44	1.81
Tensile Strength (MPa)	2920	4900
Elastic Modulus (GPa)	70.5	250
Elongation at Break (%)	3.6	2.0

**Table 2:** Planning of HFRC specimens.

Experiment	Fiber Mix-Proportion	HFRC (Kevlar 12 mm/ Carbon 24 mm)	HFRC (Carbon 12 mm/ Kevlar 24 mm)	Benchmark	Total
Compressive Test	100–0%	3	3	3	45
	80–20%	3	3		
	60–40%	3	3		
	50–50%	3	3		
	40–60%	3	3		
	20–80%	3	3		
	0–100%	3	3		
Flexural Test	100–0%	3	3	3	45
	80–20%	3	3		
	60–40%	3	3		
	50–50%	3	3		
	40–60%	3	3		
	20–80%	3	3		
	0–100%	3	3		
Splitting Tensile Test	50–50%	3	3	3	9
Impact Test	50–50%	20	20	20	60

**Table 3:** Slump values of benchmark and FRC with 1% weight percentage.

Addition of Chopped Fiber	0	1% Kevlar ^®^ 29 Fiber	1% Carbon Fiber
Slump (mm)	230	70	80

**Table 4:** Compressive strengths of benchmark and Kevlar/carbon HFRC specimens.

Specimen	Benchmark	Kevlar/Carbon HFRC Specimen (L12 mm/L24 mm)						
	C-B	C-K10/C0	C-K8/C2	C-K6/C4	C-K5/C5	C-K4/C6	C-K2/C8	C-K0/C10
**Compressive Strength (MPa)**	22.13	29.60	30.77	32.13	31.97	30.25	29.01	27.26
	23.97	31.54	32.64	33.01	33.14	31.11	29.19	28.60
	23.98	31.61	33.11	33.60	33.24	32.72	29.98	28.83
**Average** **Compressive Strength (MPa)**	23.36	30.92	32.18	32.91	32.78	31.36	29.39	28.23
**Increase (%)**	-	32	28	41	40	34	26	21

**Table 5:** Compressive strengths of benchmark and carbon/Kevlar HFRC specimens.

Specimen	Benchmark	Carbon/Kevlar HFRC Specimen (L12 mm/L24 mm)						
	C-B	C-C10/K0	C-C8/K2	C-C6/K4	C-C5/K5	C-C4/K6	C-C2/K8	C-C0/K10
**Compressive Strength (MPa)**	22.13	32.44	31.59	33.90	32.39	29.31	30.10	29.68
	23.97	33.07	31.95	34.65	32.80	30.55	30.33	29.98
	23.98	33.65	32.31	35.13	32.88	32.36	31.12	32.21
**Average** **Compressive Strength (MPa)**	23.36	33.06	31.95	34.56	32.69	30.74	30.52	30.62
**Increase (%)**	-	41	37	48	40	32	31	31

**Table 6:** Flexural strengths of benchmark and Kevlar/carbon HFRC specimens.

Specimen	Benchmark	Kevlar/Carbon HFRC Specimen (L12 mm/L24 mm)						
	F-B	F-K10/C0	F-K8/C2	F-K6/C4	F-K5/C5	F-K4/C6	F-K2/C8	F-K0/C10
**Flexural Strength (MPa)**	4.90	6.17	6.45	6.52	7.03	7.35	6.60	6.60
	4.94	6.24	6.48	6.66	7.18	7.55	6.70	6.70
	5.02	6.26	6.49	6.69	7.52	7.59	6.75	6.75
**Average** **Flexural Strength (MPa)**	4.92	6.22	6.47	6.62	7.25	7.50	6.68	6.97
**Increase (%)**	-	26	31	34	46	51	35	41

**Table 7:** Flexural strength of benchmark and Kevlar/carbon HFRC specimens.

Specimen	Benchmark	Kevlar/Carbon HFRC Specimen (L12 mm/L24 mm)						
	F-B	F-K10/C0	F-K8/C2	F-K6/C4	F-K5/C5	F-K4/C6	F-K2/C8	F-K0/C10
**Flexural Strength (MPa)**	4.90	6.39	6.54	6.62	7.00	6.30	6.02	6.01
	4.94	6.43	6.58	6.92	7.25	6.43	6.05	6.07
	5.02	6.45	6.65	7.21	7.28	6.54	6.07	6.14
**Average** **Flexural Strength (MPa)**	4.92	6.42	6.59	6.92	7.18	6.42	6.05	6.07
**Increase (%)**	-	30	33	40	45	30	22	23

**Table 8:** Splitting tensile strengths of benchmark and HFRC specimens.

Specimen	Benchmark	HFRC Specimen (L12 mm/L24 mm)	
	S-B	S-K5/C5	S-C5/K5
**Splitting Tensile Strength (MPa)**	2.33	3.09	2.96
	2.46	3.16	2.99
	2.48	3.21	2.99
**Average Splitting Tensile Strength (MPa)**	2.42	3.15	2.98
**Increase (%)**	-	30	23

**Table 9:** Impact energy-number relationship of benchmark and HFRC specimens.

Specimen	Impact Energy (J)	Impact Number				Average Impact Number	Increase (%)
I-B	150	1	1	1	2	1.25	-
	125	2	2	2	2	2	-
	100	3	4	4	4	3.75	-
	75	7	7	8	8	7.5	-
	50	12	14	14	15	13.75	-
I-K5/C5 (L12 mm/L24 mm)	150	4	4	4	5	4.25	244
	125	10	13	14	16	13.25	565
	100	43	46	53	61	50.75	1,255
	75	112	128	136	147	130.75	1644
	50	512	532	551	558	538.25	3815
I-C5/K5 (L12 mm/L24 mm)	150	3	3	4	4	2.75	124
	125	7	8	9	11	8.75	170
	100	31	35	39	42	36.75	868
	75	97	106	122	128	113.25	1411
	50	432	477	483	503	473.75	3346

## Data Availability

Not applicable.
